# High Prevalence of *Helicobacter pylori* Infection Among School-Aged Children in Ho Chi Minh City, VietNam

**DOI:** 10.3389/ijph.2022.1605354

**Published:** 2022-11-10

**Authors:** Thai Hoang Che, Tu Cam Nguyen, Dung Thi Thuy Ngo, Hiep Thanh Nguyen, Khang Tan Vo, Xuan Minh Ngo, Dinh Quang Truong, Patrick Bontems, Annie Robert, Phuong Ngoc Van Nguyen

**Affiliations:** ^1^ Department of Biostatistics and Informatics, Faculty of Public Health, Pham Ngoc Thach University of Medicine, Ho Chi Minh City, Vietnam; ^2^ Pôle Epidémiologie et Biostatistique (EPID), Institut de Recherche Expérimentale et Clinique (IREC), Faculté de Santé Publique (FSP), Université Catholique de Louvain, Brussels, Belgium; ^3^ Department of Gastroenterology and Hepatology, City Children’s Hospital, Ho Chi Minh, Vietnam; ^4^ Department of Epidemiology, Pham Ngoc Thach University of Medicine, Ho Chi Minh City, Vietnam; ^5^ Faculty of Public Health, Pham Ngoc Thach University of Medicine, Ho Chi Minh City, Vietnam; ^6^ Department of Physiology, Pathophysiology and Immunology, Pham Ngoc Thach University of Medicine, Ho Chi Minh City, Vietnam; ^7^ Faculty of Medicine, University of Medicine Pham Ngoc Thach, Ho Chi Minh City, Vietnam; ^8^ Department of Surgery, City Children’s Hospital, Ho Chi Minh, Vietnam; ^9^ Gastroenterology, Hôpital Universitaire des Enfants Reine Fabiola, Université libre de Bruxelles, Brussels, Belgium

**Keywords:** Vietnam, prevalence, school-aged children, *Helicobacter pylori*, pupils, Ho Chi Minh City

## Abstract

**Objectives:** There is no study on *Helicobacter pylori* (*H. pylori*) infection in pupils of Ho Chi Minh city (HCMC), the most overcrowded city in Vietnam. Therefore, the aim of this study was to estimate the prevalence of *H. pylori* and its geographical spread among school-aged children.

**Methods:** A school-based cross-sectional study was conducted among 1854 pupils across 24 districts of HCMC in 2019. Multiple-stage sampling method was used to enroll pupils. We built a four-points index for geographical division based on population density and employees density to evaluate the link between *H. pylori* and crowded level. Stool samples were analyzed by monoclonal enzyme-immunoassay stool antigen-test to assess the infection status. Logistic regression was performed to assess possible factors related to *H. pylori* infection.

**Results:** The overall prevalence of *H. pylori* was 87.7%. There was a linear increasing trend in the infection rate (*p* < 0.001) across the 4-points index of HCMC and this trend maintained within both age and gender subgroups (*p* = 0.02).

**Conclusion:** Prevalence of *H. pylori* was high and it increased with population density or employees density. Therefore, it is crucial to plan and implement the reduction of *H. pylori* infection programs by targeting the highly concentrated population areas of HCMC.

## Introduction

*Helicobacter pylori* (*H. pylori*) infection is a common chronic infection, affecting more than 50% of the world’s population [1]. The prevalence has changed over the last 10 years, showing a decrease in developed countries but remaining high in most developing countries [1, 2]. Like other developing countries in Southeast Asia, the prevalence of *H. pylori* in Vietnam remains high. A recent study in Hanoi in the North of Vietnam showed that the prevalence of the infection was 76.8% [3]. There was no such community-based prevalence study on *H. pylori* infection in Ho Chi Minh City (HCMC), despite it is one of the most overcrowded cities globally with a population of 8.933.082 in total and a population density of 12,000 p/km^2^. Its population density is five times higher than Hanoi (2,455 p/km^2^), three times higher than Shanghai city (3,800 p/km^2^), eighty times higher than the average of Asia (150 p/km^2^) [4, 5]. Furthermore, the link between *H. pylori* infection and crowded living conditions has been shown in several studies [6, 7]. Therefore, assessing the prevalence of *H. pylori* in HCMC is an essential public health issue in HCMC.

*H. pylori* infection is usually acquired during childhood and tends to persist if untreated [8–10]. *H. pylori* infection is a risk for duodenal-gastric ulcers, gastric cancer, and its treatment has been proved to decrease cancer risk in individuals with a family history of gastric cancer [11, 12]. Therefore, reducing *H. pylori* infection rate in the young population is crucial for decreasing the burden of gastric cancers in future.

We therefore conducted a study to estimate the prevalence of *H. pylori* infection and its geographical spread in school-aged children across the 24 districts of HCMC, Vietnam.

## Methods

### Study Design

The present school-based cross-sectional study was conducted across the 24 districts of HCMC. Two education systems co-exist in HCMC; public schools represent 96.7% (*n* = 761) and private schools account for 3.3% (*n* = 26) [13–15]. Our study was conducted across the public schools system of HCMC, which consists of 491 primary schools with grade 1st–5th (6–11 years) and 270 secondary schools with grade 6th–9th (12–15 years), representing a total of 1,077,105 pupils [14].

In order to estimate the prevalence with a precision of 7.5%, a size of 206 pupils is needed for a prior prevalence of 76.8%, as that observed in Hanoi, assuming a loss rate of 20%, and using a cluster design effect of 1.4. With 9 grades, this led to a size of 1854 pupils, or 9 pupils per class if there are 216 classes.

In each of 24 districts, we selected at random one primary school and the closest secondary school. In each of these 48 schools, one class per grade was randomly selected among the 10 to 14 classes within the grade, leading to a total number of 216 classes. In each class, 9 pupils were randomly chosen within the sequential list of inscriptions and were invited to participate into the study.

### Eligibility Criteria

Eligibility criteria included healthy school-aged children of both sexes, attending primary (6–11 years of age) and secondary (12–15 years of age) public schools in HCMC. Excluded criteria were a history of gastrointestinal endoscopy or surgery, previous *H. pylori* infection, a treatment with antibiotics or with a proton pump inhibitor (PPI) within the last 4 weeks, a treatment with a bismuth-containing compound within the last 2 weeks. Pupils diagnosed with an acute or chronic gastrointestinal disorders were also excluded.

### A Four-points Index for a Geographical Division of HCMC According the Crowd

**FIGURE 1:**
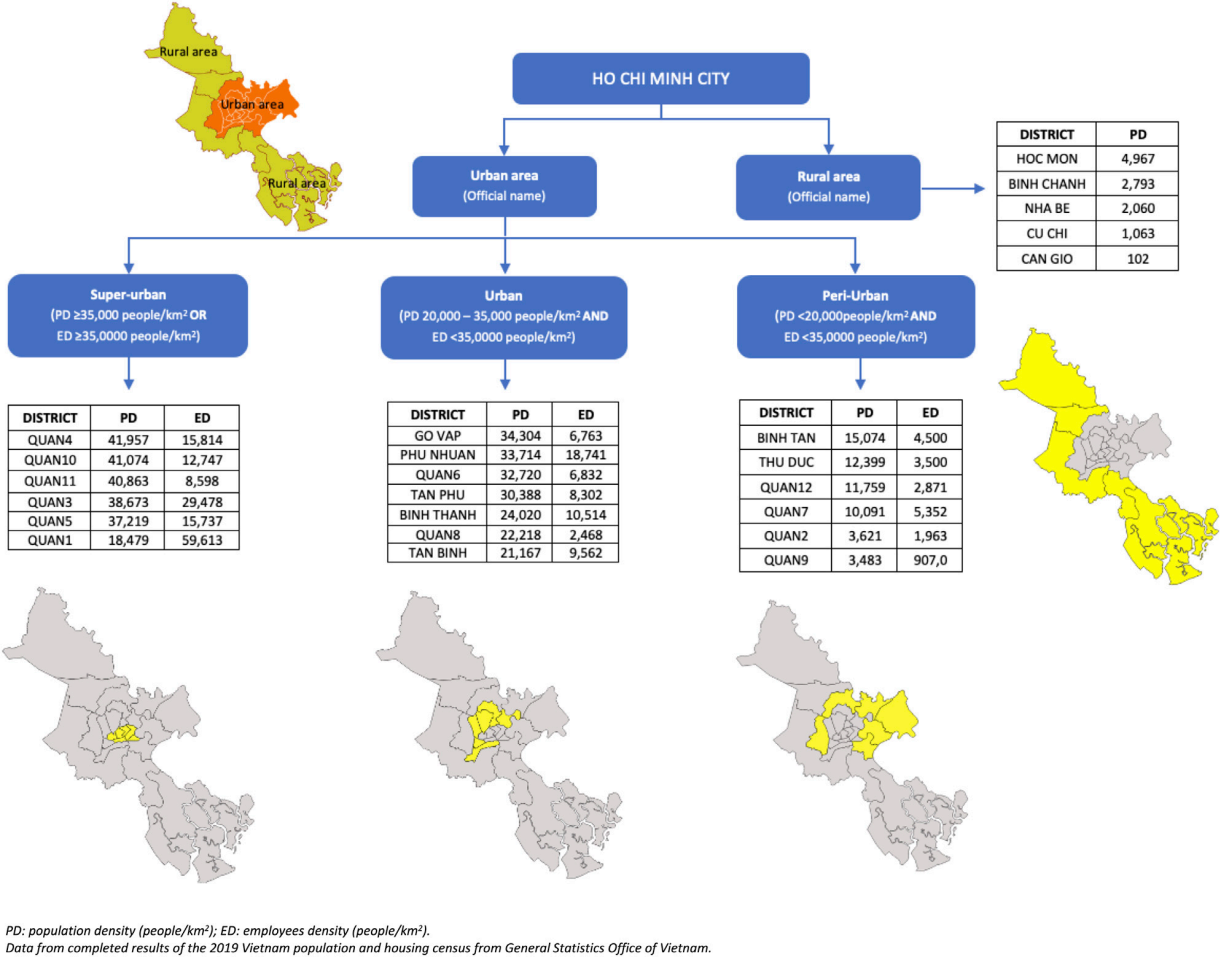
The 4-points index for the geographical division of Ho Chi Minh city (Ho Chi Minh City, Vietnam. 2019).

To assess a potential link between *H. pylori* infection and the crowded level of HCMC, we built a four-points index for the geographical division of HCMC based on population density (PD) and employees density (ED) as illustrated on Figure 1. The city is administratively divided into 24 districts, comprising 5 rural districts and 19 urban districts. Rural area was kept as the official definition, included BINH CHANH, CAN GIO, CU CHI, HOCMON, and NHA BE districts. We divided urban districts into three sub-areas: peri-urban area, urban area, and super-urban area. Urban districts with PD below 20,000 p/km^2^ and ED below 35,000 p/km^2^ were classified as peri-urban areas; it covered QUAN2, QUAN7, QUAN9, QUAN12, BINH TAN, and THU DUC districts. Districts with PD between 20,000 and 35,000 p/km^2^ and ED below 35,000 p/km^2^ were classified as urban area; it covered 7 districts: QUAN6, QUAN8, BINH THANH, GO VAP, PHU NHUAN, TAN BINH, and TAN PHU. Districts with a PD above 35,000 p/km^2^ or an ED above 35,000 p/km^2^ were classified as super-urban area; it corresponded to 6 districts: QUAN1, QUAN3, QUAN4, QUAN5, QUAN10, and QUAN11.

### Data Collection

A standard questionnaire was used to collect relevant data on *H. pylori*-related factors. The questionnaire was filled by pupils and their parents under the instructions of well-trained interviewers and researchers in the classroom. Data was entered into Microsoft Excel sheets by two independent researchers for cross-checking validation. Any discrepancy was resolved by another trained staff based on paperback documents.

### Assess *H. pylori* Infection Status

The positive status of *H. pylori* infection was confirmed by using a monoclonal enzyme-immunoassay (EIA) stool antigen test, Premier Platinum HpSA Plus test (manufactured by Meridian Bioscience, USA). The stool samples were collected and analyzed following the manufacturer’s instructions and guidelines [16]. Stool specimens were excluded if there was any water or urine in the sample. Results were classified as positive if the cut-off value of optical density (OD) was equal to or greater than 0.100, as recommended by manufacturer.

### Statistical Analysis

Maps of *H. pylori* infection prevalence were drawn using QGIS 3.16 for Mac. Statistical analyses were performed using Stata 17.0/IC software for Mac (TX: StataCorp LP). We report number with percentage for categorical variables and mean ± standard deviation for continuous variables. The demographic characteristics of pupils were compared using a Person Chi2 test for categorical variables and a Student’s t-test for continuous variables. Trends across ordered categories were tested using the Cochran-Armitage chi-square test. Interactions between the crowd index and age or sex were tested using likelihood ratio chi-square test and using Akaike’s information criterion. Logistic regression analysis was performed to assess the independent contribution of each factor to *H. pylori* infection. The significance level for all tests was set to 0.05.

### Ethical Considerations

Written informed consent was obtained from both parents (legal guardians) and pupils, who were also informed that participating in the survey was voluntary. All collected data were stored anonymously and used for research purposes only. The study protocol was approved by the Ethical Review Committee and the Scientific Committee of the University of Medicine Pham Ngoc Thach, and the Ethical Review Committee of Université catholique de Louvain—Brussels campus in Belgium.

## Results

A total of 1854 pupils were invited to participate but 20.3% refused. This refusal rate was similar across districts. Eighteen pupils were excluded (4 children who did not perform the stool test, 5 children were using antibiotics in the last 4 weeks and 9 children had a missing age or gender). The remaining 1,460 pupils were included in the present analysis. Of these, 730 (50%) were boys, and the mean age was 10.1 ± 2.7 years with a range of 6–15 years (Table 1).

**TABLE 1:** Characteristics of children in the study (Ho Chi Minh City, Vietnam. 2019).

Variables	Total
	*n* = 1,460
Age (years)	10.1 ± 2.7
6–8	483 (33.1)
9–11	473 (32.4)
≥12	504 (34.5)
Gender	
Boy	730 (50)
Girls	730 (50)
Living area	
Super-urban area	317 (21.7)
Urban area	495 (33.9)
Peri-urban area	356 (24.4)
Rural area	292 (20.0)

Values expressed means ± standard deviation or numbers (percentage).

**FIGURE 2:**
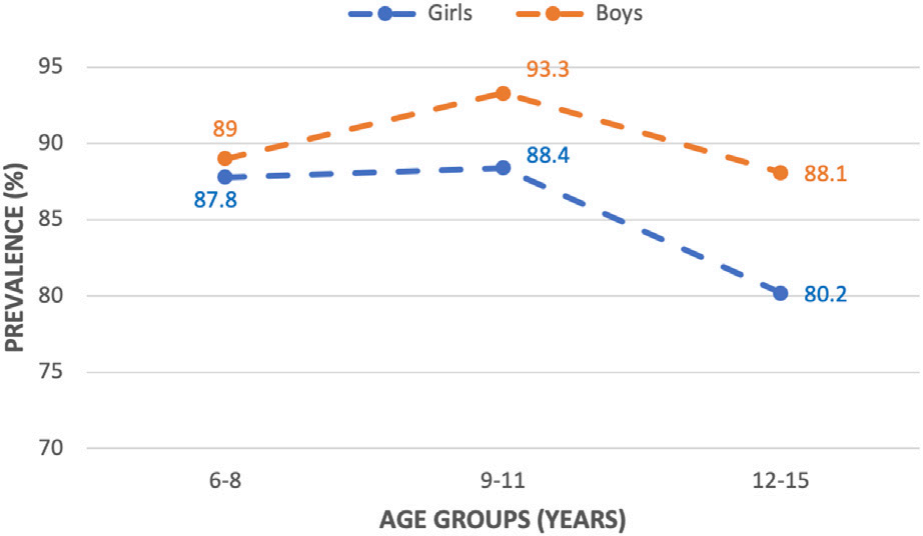
The prevalence of *Helicobacter pylori* according to age groups and gender (Ho Chi Minh City, Vietnam. 2019).

The overall prevalence of *H. pylori* infection was 87.7% (1,280/1,460). The prevalence was significantly higher in boys (90.0%, χ2 test with *p* = 0.003), and in children aged 9–11 years (90.7%, χ2 test with *p* = 0.007). The prevalence of *H. pylori* infection according to the age groups and gender is reported in Figure 2. In both sexes, the prevalence increased with age, peaked in the 9–11 age group, and then decreased when the child was 12 years or more.

**FIGURE 3:**
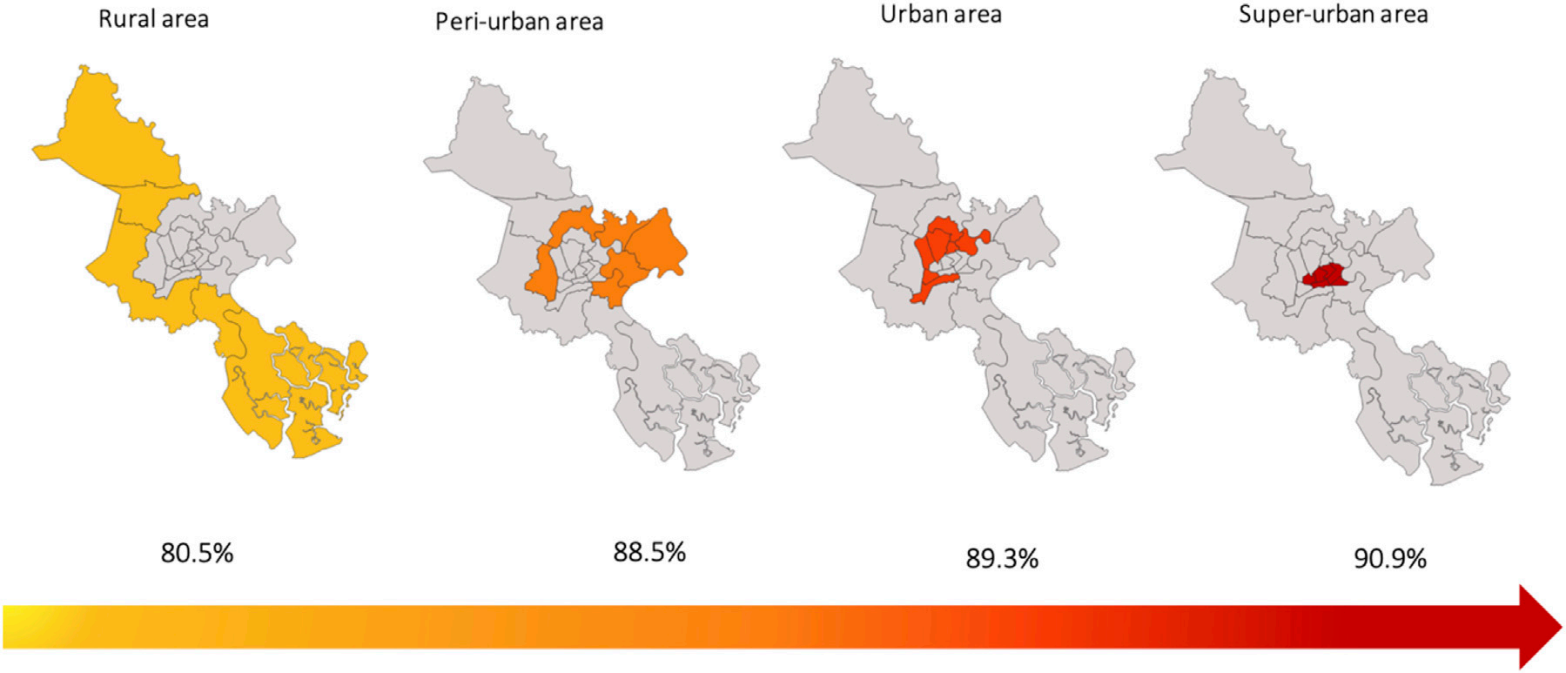
Mapping of the prevalence of *Helicobacter pylori* infection in Ho Chi Minh City according to the 4-points index (Ho Chi Minh City, Vietnam. 2019).

Figure 3 illustrates a linear increasing trend in the prevalence of *H. pylori* infection across the four-points index of crowd in HCMC (80.5% in the rural area, 88.5% in the peri-urban area, 89.3% in the urban area, 90.9% in the super-urban area, Cochran χ2 test with *p* < 0.001).

**FIGURE 4:**
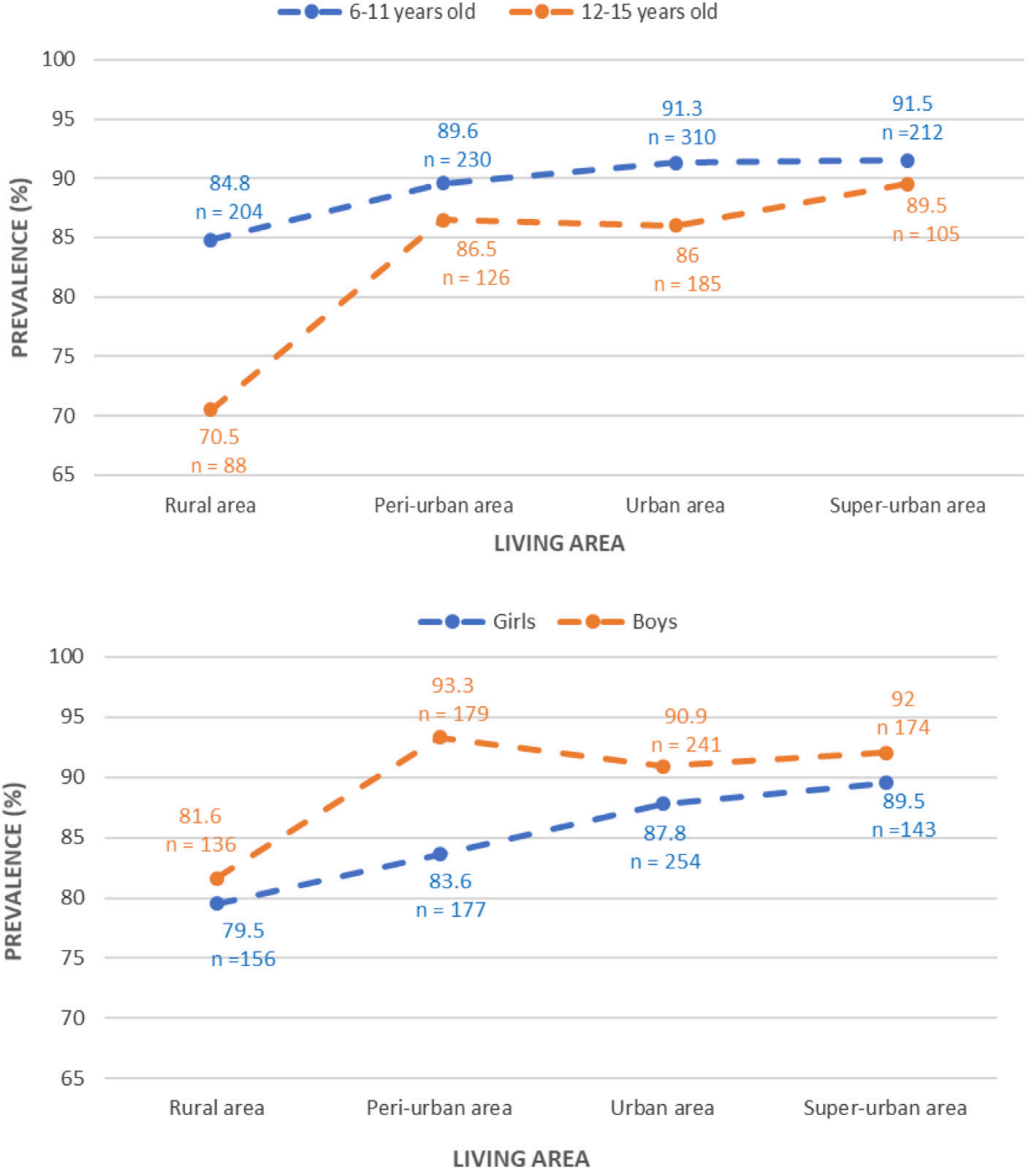
Trend in prevalence of *Helicobacter pylori* infection across the crowdy index in Ho Chi Minh City within age and gender subgroups (Ho Chi Minh City, Vietnam. 2019).

There was no interactions between the four points index and age group or gender by using likelihood ratio χ2 test (*p* = 0.64). When splitting according to age subgroups and to gender, this increasing linear trend maintained (Figure 4). Prevalence of *H. pylori* infection increased significantly as PD or PE increased within both age and gender subgroups (*p* = 0.02).

Table 2 shows the results from the logistic regression performed to assess demographic factors associated with *H. pylori* infection. Multiple logistic regression analysis showed that age, gender, and living area were significantly related to *H. pylori* infection. The prevalence of *H. pylori* was significantly increased in children aged 9–11 years [OR = 1.91, 95% Cl: 1.29–2.85, *p* = 0.003] and in boys [OR = 1.5, 95% Cl: 1.08–2.06, *p* = 0.015]. A multivariate analysis also clearly showed that the prevalence of *H. pylori* infection significantly increased with the level of crowded: PD < 20,000 p/km^2^ and ED < 35,000 p/km^2^ [OR = 1.85; 95% Cl: 1.19–2.88, *p* < 0.001]; PD 20,000–35,000 p/km^2^ and ED < 35,000 p/km^2^ [OR = 2.05; 95% Cl: 1.10–2.09, *p* < 0.001]; and PD ≥ 35,000 p/km^2^ or ED ≥ 35,000 p/km^2^ [OR = 2.33; 95% Cl: 1.44 – 3.10, *p* < 0.001].

**TABLE 2:** Univariable and multivariable analyses for possible factors associated with *Helicobacter pylori* infection (Ho Chi Minh City, Vietnam. 2019).

			Univariate analysis		Multiple analysis	
	n	% positive	OR (95%Cl)	*p*	OR (95%Cl)	*p*
Age group (years)				0.007		0.003
6–8	483	88.4	1.43 (0.99–2.07)		1.61 (1.10–2.34)	
9–11	473	90.7	1.83 (1.24–2.72)		1.91 (1.29–2.85)	
≥12	504	84.1	1		1	
Gender				0.003		0.015
Female	730	85.3	1		1	
Male	730	90.0	1.55 (1.13–2.12)		1.50 (1.08–2.06)	
Living area				<0.001		<0.001
Super-urban area	288	90.8	2.40 (1.49–3.89)		2.33 (1.44–3.10)	
Urban area	283	87.6	2.00 (1.35–3.04)		2.05 (1.10–2.09)	
Peri-urban area	227	86.3	1.86 (1.21–2.89)		1.85 (1.19–2.88)	
Rural area	235	80.5	1		1	

## Discussion

Our study is the first research that reports the current prevalence of *H. pylori* infection among school-aged children in HCMC. The overall prevalence of *H. pylori* in the representative sample of pupils in HCMC was 87.7%. Several studies on the prevalence of *H. pylori* infection in Vietnamese children ranged from 32.1% in the Mekong region [17] to 55.5% in Nghe An [18]. The prevalence observed in our study was higher compared with those reported in the previous studies, reflecting the increasing trend of *H. pylori* infection over the past decades. However, most of those previous studies were conducted in the countryside or mountainous areas, had a small sample size, and did not address all school-aged children. Moreover, all previous reports used sera diagnostic methods known to have low accuracy and could not differentiate a lifetime infection from an active infection. While in our study, all pupils at all grades in primary and secondary schools in 24 districts of HCMC were recruited. In addition, the Premier Platinum HpSA Plus stool test (Meridian Bioscience, USA) used in our study has proved a reliable tool to detect *H. pylori* infection with an accuracy of 93.4% [17, 18]. Nguyen TVH et al. conducted a validation study of the Premier Platinum HpSA PLUS test (Meridian bioscience, USA) in 232 Vietnamese children and reported a sensitivity of 97% and specificity of 95% [19]. Therefore, our results reflect an accurate infection prevalence of school-aged children in HCMC.

Several studies in Japan and Taiwan reported that *H. pylori* infection in children decreased in each age group [20–23]. In contrast, two studies from China showed that the infection increased with age [24, 25]. In our study, the prevalence of *H. pylori* infection increased with age up to 11 years, and then began to decline at 12 years of age in both boys and girls (Figure 2). These evidences suggest that the incidence rate of *H. pylori* across age groups varies greatly in different countries and may depend on different socio-economic statuses, living conditions, and lifestyle factors. Furthermore, the prevalence of *H. pylori* in HCMC was already high in the youngest ones, indicating that the acquisition of *H. pylori* occurs in very early childhood. Therefore, the building of programs to prevent and reduce the incidence of *H. pylori* has to be prioritized in public health policies.

Although HCMC takes up just 10% of the Southeast (SE) region’s land area (2,061.2/23,564.24 km^2^), it concentrates up to 50% population (8,993.082/17,828.907 inhabitants) of the SE region with PD of 4,292 p/km^2^ [5, 13]. However, it is widely believed that the population has been seriously underestimated. Indeed, one study was conducted to estimate the actual population of HCMC by counting motorcycles, which is the main transport in HCMC and 90% of household-owned motorcycles, showed that the actual population was higher than three times the officially counted [4]. Therefore, the actual PD of HCMC could be up to 12,000 p/km^2^, which means HCMC is an overcrowded city. Furthermore, several studies showed that the *H. pylori* infection is related to the crowded level. In order to control for all these qualities, we decided to build a 4-points index for the geographical division of HCMC mainly based on population density to have an insight on the level of crowds across the 24 districts of the city. And this index could aid us in explaining the *H. pylori* infection distribution in HCMC. During the process, we realized that some of the city center districts had relatively low PD compared to others, despite they are the busiest districts, where most offices and buildings are located, and almost all employees come there to work every day. Therefore, using both PD and PE to define the crowdy index woud be more reliable to find the possible link between *H. pylori* infection and the crowd level of HCMC.

By using the 4-points index, our study showed that there was a linear increasing trend in the prevalence of the infection across four areas in HCMC (Figure 3), and its trend was also found in both age or genders subgroup (Figure 4). Furthermore, the crowded index did not correlate with age group and gender by using likelihood ratio chi-square test (*p* = 0.64). That means the prevalence significantly increased as population density or employees density increased and is the same way whatever the age or gender subgroup. Indeed, a study performed in the North of Vietnam found that the higher crowded living area was a positive risk factor for *H. pylori* infection [3]. Additionally, reports from China [24], Japan [21], Jordan [26], and Nepal [27] also observed that the infection rate significantly increased in people living in crowded areas. However, these studies stratified the living area only according to population density whereas our study developed the 4-point index based on both population density and employees density as previously described and explained. That crowdy index can create a more comprehensive understanding of the relation between *H. pylori* infection and the level of populated concentration in HCMC. Therefore, designing the programs to prevent and reduce the *H. pylori* infection in HCMC should focus on the highly concentrated population areas.

The differences in the target population, the diagnostic tests, and the cut-off values of the test cause some difficulties when comparing our results to the findings in other countries. Using the same stool antigen test, a cross-sectional study in Portugal published in 2011 reported that the prevalence of *H. pylori* infection in children (0–15 years old) was 32% [28]. The survey conducted in China, among all children aged (0–15 years) published in 2020, reported the prevalence was 32.6% [24]. Another cross-section study conducted in Thailand in 2009 reported that the prevalence in children (5–7 years old) was 44.8% [29].

At the early stage of the study, the required sample size was 1854 pupils after applying the appropriate formula, as demonstrated in the study design part. In fact, we had only 1,478 pupils were enrolled in the study, which was smaller than the initial number. However, we were assumed that the loss rate of 20% at the beginning of the study development. That means 1,483 children were required to enroll in our study. Therefore, our sample size was large enough to find a difference.

The study has several strengths included this was an all school-ages community-based study that was the first to be conducted in HCMC. The sample size was large with 1854 pupils could represent the sample of pupils of HCMC. Moreover, the stool test used to detect the infection status is highly accurate and also have validated for Vietnamese children with high sensitivity and high specificity [19].

### Conclusion

Our study reports an important public health issue of *H. pylori* infection in Ho Chi Minh City. The current feco-prevalence of *H. pylori* among school-aged children remains high and it significantly increased with population density or employees density. We therefore suggest that it is crucial to plan, implement the reduction and prevention of *H. pylori* infection programs by targeting the highly concentrated population areas in HCMC. Further analyses should be focused on the impact of behavioral factors, lifestyle factors and environmental factors of children and their families that might affect the prevalence of *H. pylori* infection.
